# Testicular steroidogenesis is suppressed during experimental autoimmune encephalomyelitis in rats

**DOI:** 10.1038/s41598-021-88305-5

**Published:** 2021-04-26

**Authors:** Ana Milosevic, Ivana Bjelobaba, Iva D. Bozic, Irena Lavrnja, Danijela Savic, Katarina Tesovic, Marija Jakovljevic, Stanko S. Stojilkovic, Marija M. Janjic

**Affiliations:** 1 Department for Neurobiology, Institute for Biological Research “Siniša Stanković”-National Institute of Republic of Serbia, University of Belgrade, Bulevar despota Stefana 142, 11000 Belgrade, Serbia; 2 Section on Cellular Signaling, Eunice Kennedy Shriver National Institute for Child Health and Human Development, National Institutes of Health, Bethesda, MD USA

**Keywords:** Immunology, Neuroscience, Physiology, Endocrinology

## Abstract

Multiple sclerosis (MS) is an autoimmune disease that usually occurs during the reproductive years in both sexes. Many male patients with MS show lower blood testosterone levels, which was also observed in male rats during experimental autoimmune encephalomyelitis (EAE), an animal model of MS. To better understand the causes of decreased testosterone production during EAE, we investigated the expression status of genes and proteins associated with steroidogenesis in the testes. No changes in the number of interstitial cells were observed in EAE animals, but the expression of the insulin-like 3 gene was reduced at the peak of the disease, implying that the Leydig cell functional capacity was affected. Consistent with this finding, the expression of most steroidogenic enzyme genes and proteins was reduced during EAE, including StAR, CYP11A1, CYP17A1 and HSD3B. No signs of testicular inflammation were observed. Recovery of steroidogenesis was observed after injection of hCG, the placental gonadotropin, or buserelin acetate, a gonadotropin-releasing hormone analogue, at the peak of EAE. Together, our results are consistent with the hypothesis that impaired testicular steroidogenesis originates upstream of the testes and that low serum LH is the main cause of decreased testosterone levels during EAE.

## Introduction

Multiple sclerosis (MS) is an autoimmune, neurodegenerative disease of the central nervous system. This chronic inflammatory disease is more common in women than in men^1^, but clinical progression tends to be faster and more severe in men^2^. Studies show that low testosterone levels in prenatal period, puberty and adulthood increase the risk for MS in men^3^. It has also been shown that low serum testosterone levels can be detected in up to 40% of MS patients^4–6^. These patients may experience secondary hypogonadism and poorer clinical outcomes, including a faster decline in cognitive abilities^7^. Similarly, we and others have shown decreased serum testosterone levels during experimental autoimmune encephalomyelitis (EAE), an animal model of MS^4,8,9^. However, the possible causes of declining testosterone levels in EAE animals or patients with MS have not yet been elucidated.

The main site of testosterone synthesis in males are testes, i.e. Leydig cells (LCs) of the testis interstitium, while a smaller amount of testosterone is also produced by the adrenal glands. Testosterone biosynthesis is mainly regulated by luteinizing hormone (LH), which is secreted from the pituitary gland in response to gonadotropin-releasing hormone (GnRH), a hypothalamic decapeptide. LH acts directly on LCs by binding to a specific high-affinity receptor on their surface, the LH receptor (LHR). Binding of LH to LHR activates the cyclic adenosine monophosphate (cAMP)/protein kinase A signaling pathway^10^.

For the synthesis of steroid hormones, LCs use cholesterol from various sources. Bulk delivery of cholesterol esters to LCs is mediated by “selective” extracellular cholesterol uptake that uses class B type I scavenger receptors (SCARB1)^11^. A rate-limiting and hormone-sensitive step in steroidogenesis is the transport of cholesterol from the outer to the inner mitochondrial membrane, through the aqueous intra-membrane space. The acute phase of steroidogenesis, represented by increased mobilization and transport of cholesterol to the inner mitochondrial membrane, occurs within a few minutes after LH binding to its receptor. This phase is determined by the action of steroidogenic acute regulatory protein (StAR)^12,13^.

The process of steroidogenesis involves two major enzyme groups: cytochrome P450 (CYPs), which catalyzes hydroxylation and cleavage reaction of steroid substrates, and hydroxysteroid dehydrogenases (HSDs), involved in steroid hormone reduction and oxidation reactions. These enzymes are found on the inner mitochondrial and endoplasmic reticulum membranes. The enzymatic phase of steroidogenesis begins with the conversion of cholesterol to pregnenolone and isocaproaldehyde, catalyzed by cholesterol side-chain cleavage enzyme (CYP11A1), also known as P450scc. The resulting pregnenolone is further metabolized to progesterone by the mitochondrial or microsomal enzyme 3 beta-hydroxysteroid dehydrogenase/delta 5–4 isomerase (HSD3B). In rat LCs, the enzyme steroid 17-alpha-hydroxylase/17,20 lyase (CYP17A1) catalyzes the conversion of progesterone to Δ4-androstendione. The last step in the pathway of testosterone biosynthesis is the conversion of Δ4-androstendione to testosterone by the action of 17-beta-hydroxysteroid dehydrogenase (HSD17B). The chronic phase of steroidogenesis that occurs within a few hours involves mechanisms that increase transcription/translation of these steroidogenic enzymes^14,15^.

Testosterone anti-inflammatory, neuroprotective, and promyelinating effects have been well documented using a variety of animal models and cell cultures^16^. Moreover, testosterone is considered a protective factor in autoimmune diseases, including MS and EAE^17–19^. It is therefore of great importance to clarify the events that lead to a decrease in testosterone in autoimmunity. To address this issue, we have studied the expression profile of testicular steroidogenic pathway and evaluated testicular responsiveness to in vivo application of the placental gonadotropin, hCG (human chorionic gonadotropin) and the GnRH analogue, buserelin acetate, during EAE. Our results are consistent with the hypothesis that impaired steroidogenesis in the testes originates from the upstream part of the hypothalamic-pituitary–testicular axis.

## Methods

### Animals

The experiments in this study were performed on male Dark Agouti rats, 9–12-week-old, bred in the animal facility of the Institute for Biological Research “Sinisa Stankovic”. Animals were randomly distributed prior to the experiments and housed 3–4 per cage in standard environment (12 h light/dark cycle, constant temperature and humidity, free access to laboratory chow and water). All experimental procedures were performed in accordance with European Ethical Normative (Directive 2010/63/EU) on the protection of animals used for experimental and other scientific purposes, as well as in accordance with national regulative given by the Law on Animal Welfare of the Republic of Serbia ("Official Gazette of Republic of Serbia" No. 41/2009). Experimental protocols were approved by the national licensing committee at the Department of Animal Welfare, Veterinary Directorate, Ministry of Agriculture, Forestry and Water Management of Republic of Serbia (permit no. 323-07-05970/2020-05). All methods were carried out in accordance with relevant guidelines and regulations. The ARRIVE guidelines were followed for reporting results.

### Induction and assessment of EAE

EAE was induced as previously described^9^. Animals were examined and weighed every morning for 28 days after immunization, and given grades using a scale that quantifies EAE symptoms as follows: 0—unaffected, 1—tail atony, 2—hind limb weakness, 3—total hind limb paralysis, 4—moribund state, with the score of 4 set as a humane endpoint. During the paralysis, the animals were given water by hand, and the chow was placed in accessible places. Animals were euthanized at *onset* (grade 1), *peak* (grade 3) and *end* (grade 0) of the disease. Naïve animals served as a control group. Six animals per group were used in each experiment.

### In vivo treatments

Testicular function was assessed at the peak of EAE. In the first of these experiments, naïve and peak EAE groups of animals were treated with human recombinant choriogonadotropin alpha (hCG i.e., Ovitrelle (6500 IU/0.5 ml; Merck, Bari, Italy)), subcutaneously. Six animals per group (control and peak of EAE) were administered with 100 IU or 10 IU of hCG and euthanized after 2 h or 24 h, respectively. Previously, these doses, paired with these time points, were shown to lead to the highest peaks in testosterone production after a single injection in intact rats^20,21^. In another experiment, naïve and peak EAE groups of rats (n = 6 animals per group) received an injection of buserelin acetate salt (Sigma-Aldrich, St. Louis, MO) diluted in saline, intraperitoneally (4 µg/animal) and euthanized after 1 h. In each of these two experiments, additional 12 saline-treated animals (6 naïve, 6 peak EAE) served as controls.

### Blood collection and hormone measurement

All animals were sacrificed by gradual asphyxia in a CO_2_ chamber. Blood was collected from the left ventricle, allowed to coagulate for 20 min and centrifuged at 3000 × *g* for 10 min to separate the sera. Testosterone concentrations were determined using the ELISA kits of the Cayman Chemical Company (Ann Arbor, MI, USA), following the instructions. Serums of animals treated with buserelin acetate and hCG were adequately diluted. Serum LH levels were measured using the Rodent ELISA kit (Endocrine Technologies Inc, Newark, CA), according to the manufacturer’s instructions.

### Tissue collection and interstitial cell isolation

Rats were perfused with cold saline and testes were isolated and weighed. One testis was used to isolate interstitial cells, as previously described^22,23^. Briefly, one decapsulated testis of each animal was placed in cold Dulbecco’s Modified Eagle Medium (DMEM; Gibco, Thermo Fisher Scientific, Waltham, MA) containing 1.5% w/v bovine serum albumin (BSA; Sigma), 1.2 mg/ml of collagenase (Sigma), 20 mM HEPES in a 50 ml falcon tube. After incubation in a shaking water bath, oscillating at 120 rpm for 15 min at 34 °C, cold 0.5% w/v BSA/DMEM supplemented with sodium bicarbonate was added and the tubes were left on ice for 5 min. To remove the seminiferous tubules, the suspension was filtered through 100 µm nylon cell strainer (Falcon, Corning Inc., Corning, NY). The cells were centrifuged at 150 × *g* for 5 min and the pellet washed with the same medium. Cells were finally resuspended in 0.1% w/v BSA/DMEM, supplemented with sodium bicarbonate and counted. For RNA isolation, 35 × 10^6^ cells/testis were used; the remainder was collected for protein isolation. Hemitestes (excised from the other testis of each animal) were placed in RNAlater RNA Stabilization Solution (Ambion, Applied Biosystems by Thermo Fisher Scientific, Waltham, MA), until RNA isolation.

### Immunohistochemistry

Rat testes were isolated and fixed in Bouin’s solution at room temperature for 48 h. After trimming, the tissue was dehydrated, embedded in paraffin, and cut on a microtome to 7 µm thick sections. Deparaffinized and permeabilized (0.1% TritonX-100 in PBS) tissue sections were immersed in 0.3% H_2_O_2_ in methanol and then blocked using 5% goat serum. To assess the number of macrophages in testicular interstitium, sections were incubated with mouse monoclonal CD68 (ED1) antibody (ab31630, Abcam plc, Cambridge, UK) at 1:100 dilution, overnight. Secondary goat anti-mouse-HRP conjugated antibody (sc-2004, Santa Cruz, Biotechnology Inc, Dallas, TX) was applied for 2 h and the staining was visualized using 3,3′-diaminobenzidine tetrahydrochloride (K3468, DAKO, Glostrup, Denmark). Some sections were hematoxylin counterstained. All sections were dehydrated, cleared, and mounted in DPX (Sigma). CD68-labeled sections from 4 control and 4 peak EAE animals were examined under a Leica DMRB microscope equipped with a camera (Leica Microsystems, Wetzlar, Germany).

Micrographs (3 sections/animal, 10 fields/section) at 10 × magnification were taken by a blinded observer and CD68-positive cells were counted using ImageJ/Fiji software package. Results are expressed as the mean number of CD68-positive cells per mm^2^ ± SEM. Photoshop CC (Version, 14.2, Adobe Systems Inc., San Jose, CA) was used to resize and edit micrographs.

### RNA isolation, reverse transcription and qRT-PCR

The RNA from interstitial cells was isolated using RNeasy Mini Kit (QIAGEN, Germany), according to the manufacturer's instructions, while the RNA from tissue samples was isolated via TRIzol method. In brief, 100 mg of hemitestes were placed into 1 ml of TRIzol Reagent (Thermo Fisher Scientific) and homogenized. Tissue homogenates were mixed with 0.2 ml chloroform, vortexed and centrifuged (12,000 × *g*, 4 °C, 15 min). The layer containing RNA was transferred to a clean tube, 0.5 ml of isopropanol was added, and the samples were frozen at − 80 °C. The following day, the samples were centrifuged (12,000 × *g*, 4 °C, 10 min) and the pellets were washed with 75% ethanol. After centrifugation (7500 × *g*, 4 °C, 5 min) the pellets containing RNA were diluted in 100 µl UltraPure Water (Gibco, Thermo Fisher Scientific). After determining the concentration and purity of the RNA, reverse transcription was performed using HighCapacity cDNA Reverse Transcription Kit (Applied Biosystems by Thermo Fisher Scientific). Quantitative Real Time PCR (qRT-PCR) analysis was done using TaqMan and/or SYBR Green chemistry and QuantStudio 3 real-time PCR system (Applied Biosystems by Thermo Fisher Scientific). The probes used for TaqMan chemistry were *Insl3* (Rn00586632_m1) and *Actb* (Rn00667869_m1) (Thermo Fisher Scientific, Rockford, IL). Table 1 shows the details on primer pairs, used for SYBR Green chemistry. The expression levels of the target genes were quantified by a comparative 2^–∆Ct^ method. β-actin (*Actb*) was chosen as the reference gene because its expression was more stable compared to *Gapdh* and *Hprt*.

**Table 1:** Primer sequences used for qRT-PCR analysis.

Primer	Sequence	Accession number
*Actb*	f: AGATTACTGCCCTGGCTCCT	NM_031144.3
	r: ACATCTGCTGGAAGGTGGAC	
*Cyp11a1*	f: ACTTCCTGAGGGAGAACGGC	NM_017286.3
	r: TCCATGTTGCCCAGCTTCTC	
*Cyp17a1*	f: GTTTCTCCCCAGACGTGGTC	NM_012753.2
	r: GGTCCGACAAGAGGCTTTGA	
*Hsd3b1/2*	f: GACAGGAGCAGGAGGGTTTGTGG	NM_001007719
	r: CTCCTTCTAACATTGTCACCTTGGCCT	
*Hsd17b3*	f: CTGCTTGTGTGCCTCGTTTG	NM_054007.1
	r: ACTGCCCATTGTCCCATTGA	
*Lhcgr*	f: TATGCTCGGAGGATGGCTCT	NM_012978.1
	r: AGCACAGATGACGACGAAGG	
*Scarb1*	f: GCTTCTGGTGCCCATCATTTAC	NM_031541.1
	r: AGCTTGGCTTCTTGCAGTACC	
*Star*	f: AGCAAGGAGAGGAAGCTATGC	NM_031558.3
	r: GGCACCACCTTACTTAGCACT	

### Protein isolation and western blot

Proteins were isolated from pooled interstitial cells of each group using RIPA buffer (Sigma), enriched with protease and phosphatase inhibitors (Thermo Fisher Scientific). Samples (40 µg in each lane) were loaded onto 10% SDS polyacrylamide gels and separated by gel electrophoresis. Proteins were transferred to a PVDF membrane (pore size 0.45 μm; Immobilon-P transfer membrane, Millipore, Darmstadt, Germany). For HSD3B protein analyses, membranes were stained with MemCode reversible protein stain kit for PVDF membranes (No. 24585, Thermo Fisher Scientific). Membranes were blocked for 1 h in 5% BSA or 5% non-fat dry milk (Blotto, sc-2325, Santa Cruz Biotechnology, Dallas, TX) in Tris-buffered saline with Tween-20, and then incubated with primary antibodies, at 4 °C overnight. The next day, membranes were incubated with secondary antibodies for 1 h. All antibodies used in Western blot analysis are listed in Table 2. Chemiluminescent signal was induced by SuperSignal West Femto Maximum Sensitivity Substrate and visualized using the iBright CL1500 Imaging System (both from Thermo Fisher Scientific). Blot images were subjected to densitometric analysis (ImageJ software package). The optical density (OD) of protein bands of interest in each lane was normalized to the OD of β-actin bands from the same lane. Normalized ODs from each group, from three experiments, are presented in relation to the control (100%).

**Table 2:** Antibodies used for Western blot.

Target	Source and class	Manufacturer
HSD3B	Rabbit, polyclonal	A gift from prof. Ian Mason^39^
CYP11A1	Rabbit, polyclonal	Protein Tech, 13,363–1-AP
StAR	Rabbit, polyclonal	A gift from prof. Douglas Stocco^12^
β-actin	Mouse, monoclonal	Sigma Aldrich, A5316
Anti-mouse IgG, HRP conjugated	Donkey, polyclonal	Santa Cruz, sc-2314
Anti-rabbit IgG, HRP conjugated	Donkey, polyclonal	Santa Cruz, sc-2313

### Statistical analysis

All statistical analyses were performed using GraphPad Prism (version 8.0.2 for Windows, GraphPad Software, La Jolla, CA; http://www.graphpad.com). Nonparametric tests, Mann–Whitney or Kruskal–Wallis test (followed by Dunn’s post hoc test), were used to compare the mean values between two or multiple groups, respectively. The significance of the difference in mean values was expressed for EAE groups compared to the control in all graphs unless indicated otherwise. The results are presented as mean ± SEM for each group of animals. Statistical differences are labeled as: *P < 0.05, **P < 0.01, ***P < 0.001 and ****P < 0.0001. The graphs were made using KaleidaGraph (version 4.1.0, Synergy Software, Reading, PA; http://www.synergy.com) and Illustrator (version 25.1.0, Adobe Systems Inc., San Jose, CA; http://www.adobe.com).

## Results

### EAE causes weight loss, accompanied with inhibition of steroidogenesis and expression of LC-specific genes

**Figure 1:**
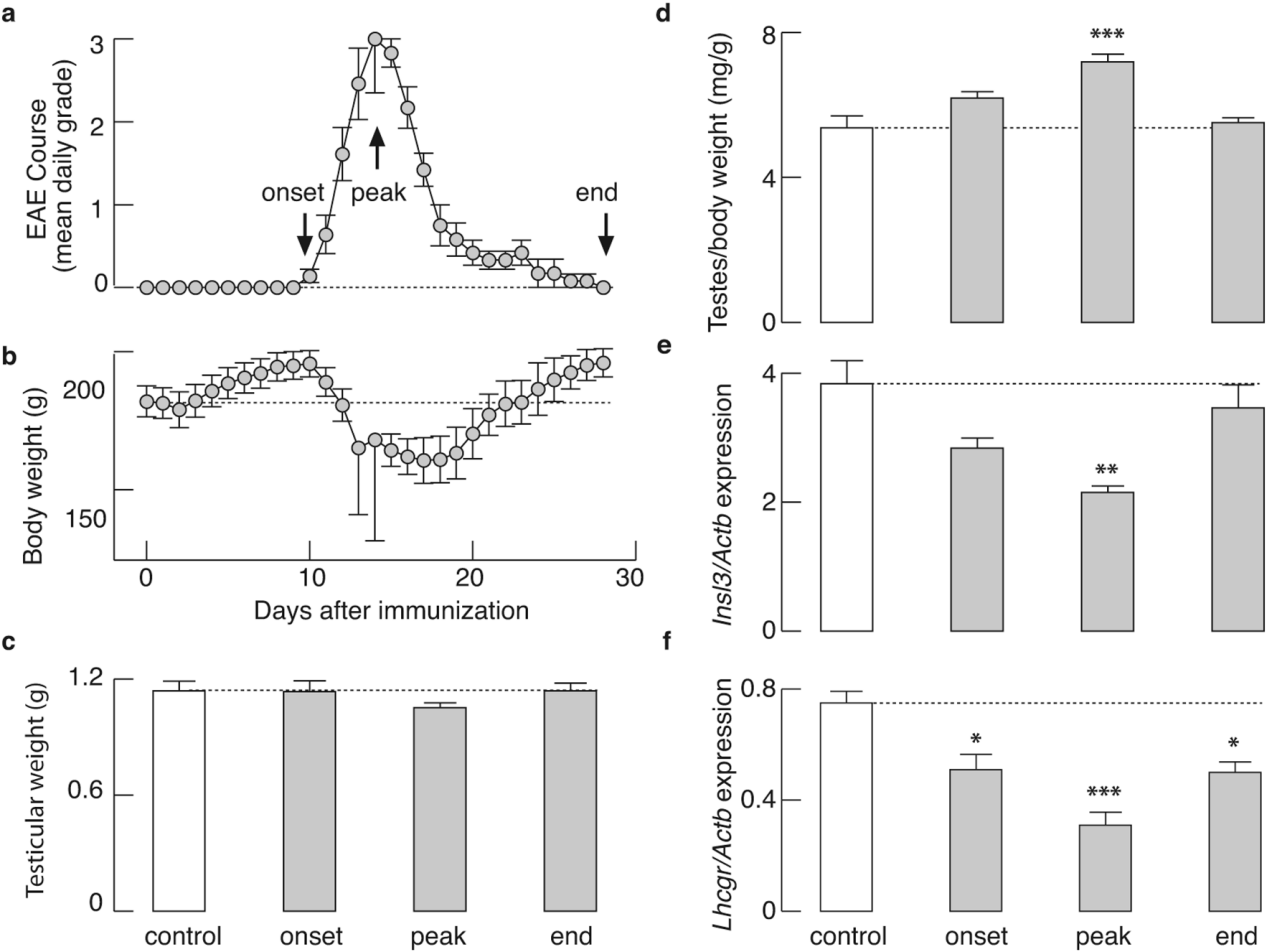
EAE course with simultaneous weight loss, without affecting testicular weight, but coinciding with changes in *Insl3* and *Lhcgr* expression in testicular tissue. (**a**,**b**) Male rats (n = 18) were graded based on the severity of EAE symptoms (**a**), and weighed (**b**) every morning for 28 days after immunization. Regardless of the body weight loss, no significant difference in testicular weight was observed, as shown on (**c**), Mean left testicular weight on the day of euthanasia for each group of animals (n = 6 per group) and (**d**), Mean ratio between testicular weight *vs.* body weight on the day of euthanasia of the same animals; (**e**) Down-regulation in the expression of *Insl3* in testicular tissue, determined by qRT-PCR, was noticeable and statistically significant at the peak of EAE, compared to control; (**f**) Down-regulation of *Lhcgr* mRNA, significant throughout the disease. The data shown are mean ± SEM (n = 6 animals/group), derived from a representative experiment. *P < 0.05, **P < 0.01, ***P < 0.001; Kruskal–Wallis test, followed by Dunn’s post hoc test. The figure was made using KaleidaGraph (version 4.1.0; http://www.synergy.com) and Illustrator (version 25.1.0; http://www.adobe.com).

Figure 1a shows the mean daily grade, while Fig. 1b represents the mean daily body weight of immunized animals (n = 18) from a representative experiment. In our hands, immunization resulted in a typical EAE course^9,24^, with symptoms peaking on day 14 after immunization (mean score 3.00 ± 0.65), and concomitant body weight loss with a nadir that coincides with the peak of the disease. In contrast, no difference in testicular weight was observed between animals from each group (control, onset, peak, end; Fig. 1c). Weight loss during EAE without an effect on testicular weight was also shown through the mean ratio of testicular weight to body weight on the day of euthanasia, calculated individually for each animal. A significant increase in this ratio was recorded at the onset (*P* < 0.05) and peak (*P* < 0.001) of EAE, compared to the control (Fig. 1d).

Consistent with our recent work^9^, serum LH and testosterone levels dropped dramatically during the onset and peak of EAE, compared with control values, but were partially restored towards the end of the disease. Serum hormone levels in the control, onset, peak, and end groups of rats were as follows (in ng/ml): LH: 3.75 ± 0.78, 0.83 ± 0.07 (*P = 0.024), 0.43 ± 0.14 (***P = 0.0009), 1.90 ± 0.56 (*P* = 0.14); testosterone: 3.63 ± 1.27, 1.46 ± 0.43 (*P* = 0.223), 0.28 ± 0.05 (**P = 0.0014), 1.74 ± 0.58 (*P* = 0.363). The mRNA expression of insulin-like 3 (*Insl3*), the biomarker of LC functionality, was also significantly downregulated (*P* < 0.01) at the peak of EAE (Fig. 1e), as well as the *Lhcgr* gene that lasted throughout the course of the disease (Fig. 1f, P < 0.01).

In contrast, the disease did not take a toll on the number of interstitial cells; no significant difference in interstitial cells numbers between control and peak EAE groups was observed (127.20 ± 15.85 × 10^6^ vs 105.70 ± 7.21 × 10^6^ cells per animal, control relative to peak EAE).

### The testicular steroidogenic pathway is down-regulated during EAE

**Figure 2:**
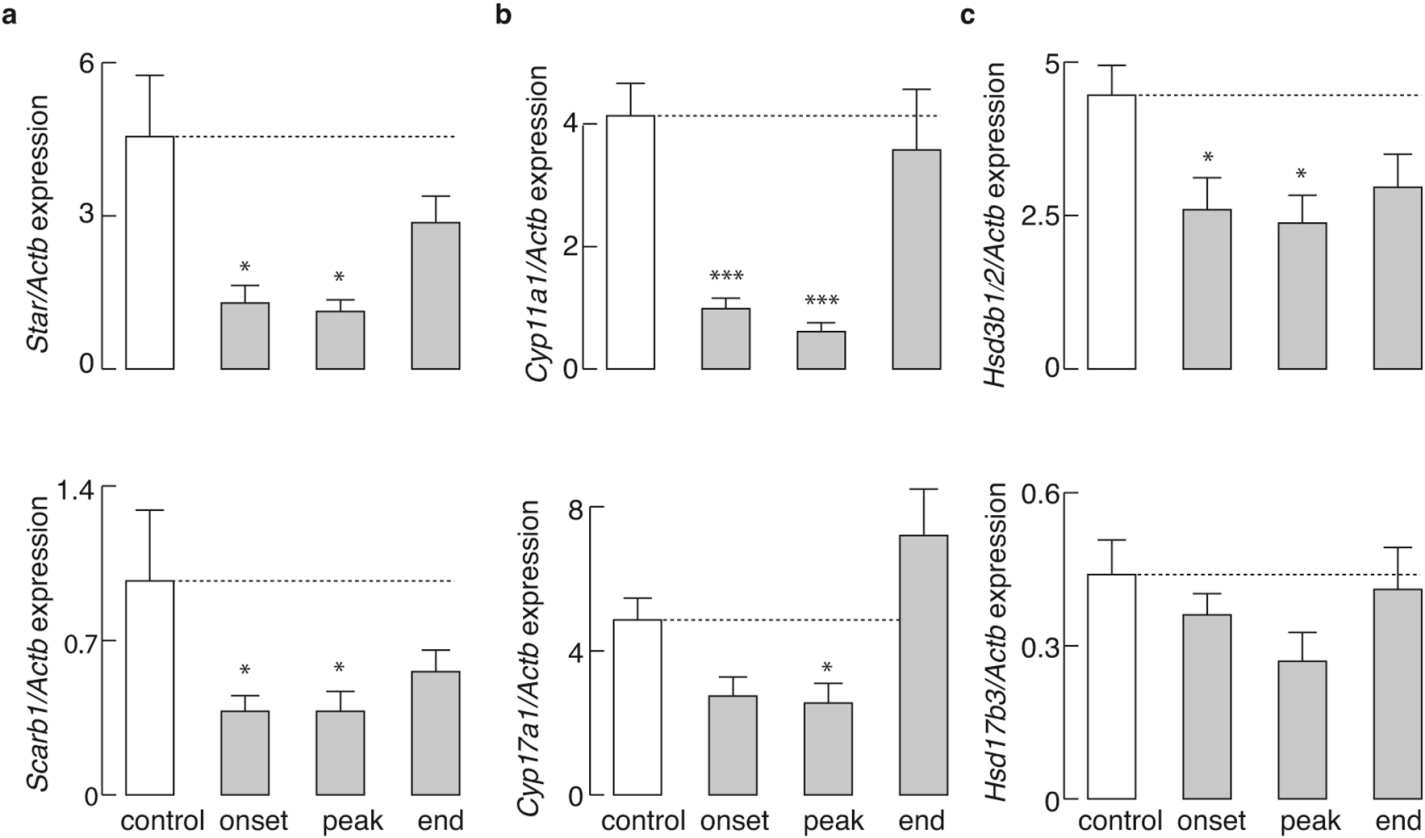
Effects of EAE on the expression profile of steroidogenic pathway genes in testicular interstitial cells. Samples isolated from interstitial cells of animals at different stages of disease and control animals (n = 6 animals /group) were subjected to qRT-PCR analysis: *Star* (**a** top), *Scarb1* (**a** bottom), *Cyp11a1* (**b** top) *Cyp17a1* (**b** bottom), *Hsd3b1/2* (**c** top) and *Hsd17b3* (**c** bottom). Plotted data are presented as mean ± SEM; * *P* < 0.05, *** *P* < 0.001, determined by Kruskal–Wallis test, followed by Dunn’s post hoc test. The figure was made using KaleidaGraph (version 4.1.0; http://www.synergy.com) and Illustrator (version 25.1.0; http://www.adobe.com).

In addition to *Lhcgr*, we also analyzed the expression of other steroidogenic pathway genes in testicular interstitial cells. The expression of *Star* and *Scarb1*, two genes that control LC cholesterol transport, was significantly reduced during the onset and peak of the disease (Fig. 2a). The mRNA expression of two members of the CYP enzyme family of genes, *Cyp11a1* and *Cyp17a1*, was similarly affected (Fig. 2b). The expression of *Hsd3b1/2* was also significantly reduced during the onset and peak of the disease, whereas the expression of *Hsd17b3*, another member of the HSD enzyme family, remained unchanged throughout the disease (Fig. 2c).

**Figure 3:**
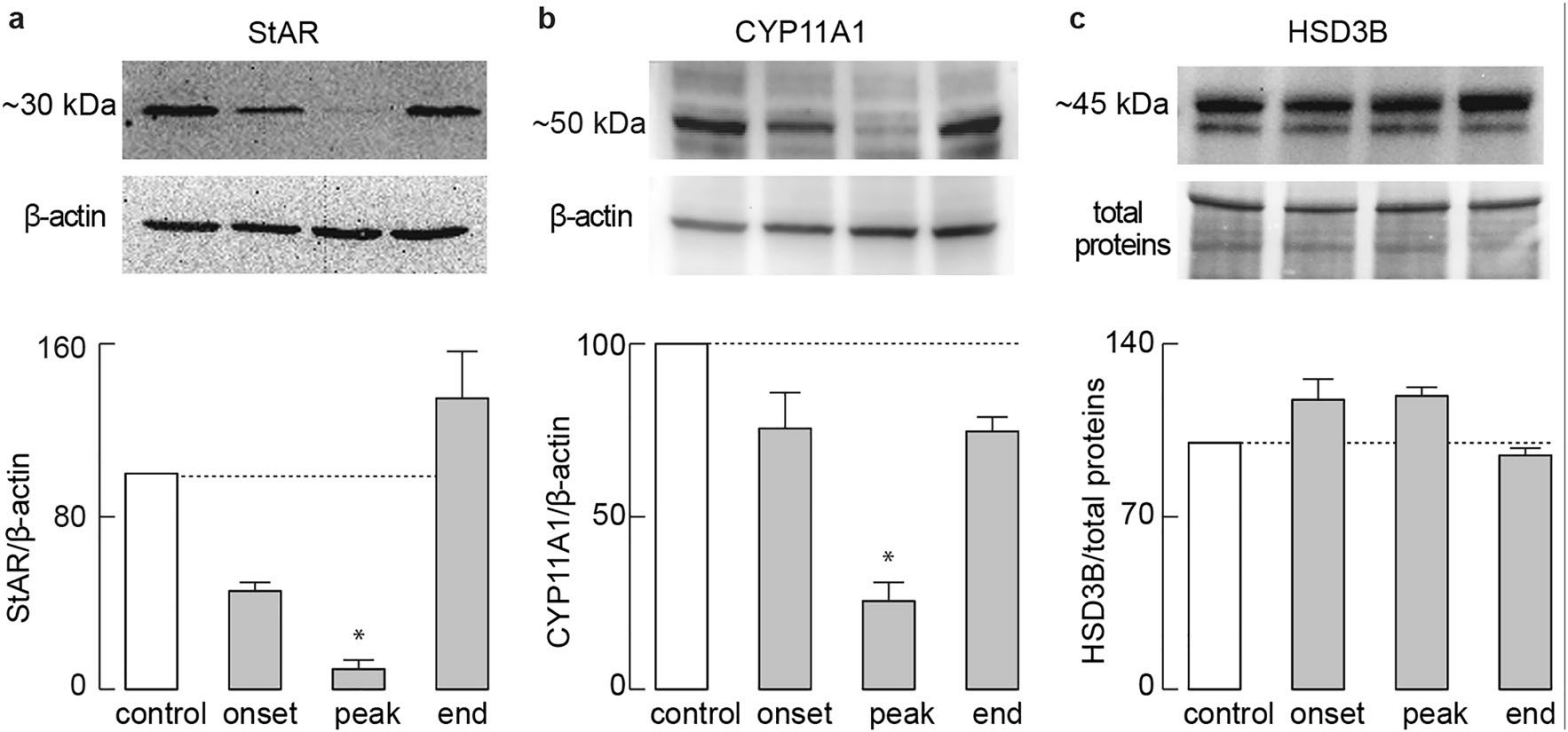
Effects of EAE on the expression profile of testicular steroidogenic pathway proteins. Pooled interstitial cells of 6 animals from each group were analyzed for StAR (**a**), CYP11A1 (**b**), and HSD3B (**c**) proteins via Western blot (40 μg proteins/well). For the quantification of HSD3B, the top bands (~ 45 kDa) were used. StAR and CYP11A1 were determined relative to β-actin, while HSD3B levels were determined relative to total protein stain. Top panels: representative blots (unprocessed images available in Supplementary figure). Bottom panels: mean ± SEM (from three Western blot experiment values), optical density values were normalized to the control group (100%).*P < 0.05, via Kruskal–Wallis test, followed by Dunn’s post hoc test. The figure was made using KaleidaGraph (version 4.1.0; http://www.synergy.com) and Illustrator (version 25.1.0; http://www.adobe.com).

We also assessed the protein levels of some components affected at mRNA levels (Fig. 3). StAR (Fig. 3a) and CYP11A1 (Fig. 3b) proteins showed a down-regulation pattern reminiscent of that in gene expression, both reaching statistical significance at the peak of EAE, while HSD3B protein levels (Fig. 3c) were not altered. Since the most prominent inhibition of steroidogenesis was observed at the peak of the disease, we used only the control and peak EAE groups of animals for experiments described below.

### EAE does not lead to testicular inflammation nor morphological changes of the seminiferous tubules

**Figure 4:**
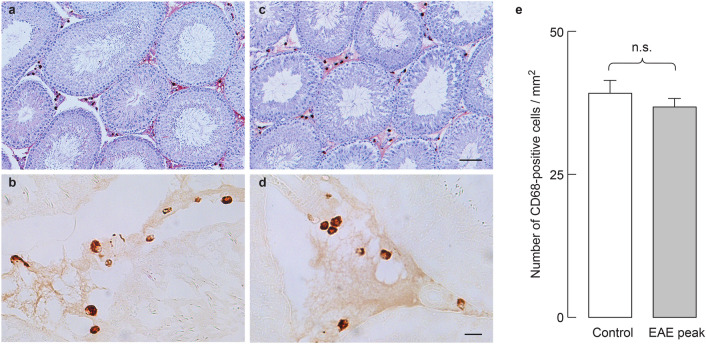
Immunohistochemical analysis of testicular macrophages in EAE. CD68 immunopositive cells were observed exclusively in the testicular interstitium of both control (**a**,**b**) and peak EAE (**c**,**d**) testes. Hematoxylin staining reveals normal histological structure of seminiferous tubules and interstitial space in both groups (**a**,**c**). Higher magnification images of the interstitium in CD68-stained sections depict macrophages in control (**b**) and peak EAE (**d**) animals. Quantification of CD68 staining (**e**) confirms that the number of macrophages does not change significantly in EAE (Mann–Whitney test). Data is plotted as mean ± SEM. Scale bar in (**c**) equals 100 μm and applies also to (**a**). Scale bar in (**d**) equals 20 μm and applies also to (**b**). The figure was made combining KaleidaGraph (version 4.1.0, http://www.synergy.com), Photochop CC (version 14.2, http://www.adobe.com) and Illustrator (version 25.1.0, http://www.adobe.com).

It is well known that pro-inflammatory cytokines can inhibit steroidogenesis by directly inhibiting the expression of steroidogenic enzyme/protein genes^25^. Since EAE is an inflammatory disease, we examined testicular inflammation during the course of this disease. The mRNA expression of the cytokines *Tnf, Ifng, Il1b* and *Il6* remained low in testicular tissue throughout the disease (data not shown). CD68 immunostaining was used to visualize macrophages, the main population of immune cells in the rat testes. Characteristic granular staining of CD68, representing newly arrived and/or transient macrophages, was present in the testicular interstitium, but not in the seminiferous tubules of the control (Fig. 4a,b) and peak EAE (Fig. 4c,d) groups of animals. Sections labeled for CD68 and counterstained with hematoxylin confirmed the absence of infiltrating mononuclear cells (Fig. 4a,c). Further analysis (Fig. 4e), implies that there is no change in the number of CD68-positive cells between the control and the peak EAE. These observations suggest that EAE-induced inhibition of steroidogenesis is not caused by testicular inflammation. Furthermore, Fig. 4a,c confirm our previous findings that spermatogenesis and spermiogenesis were not qualitatively affected during the peak of the disease^9^.

### Leydig cells respond to testicular and pituitary stimulation, but with different response amplitude in control and peak EAE animals

**Figure 5:**
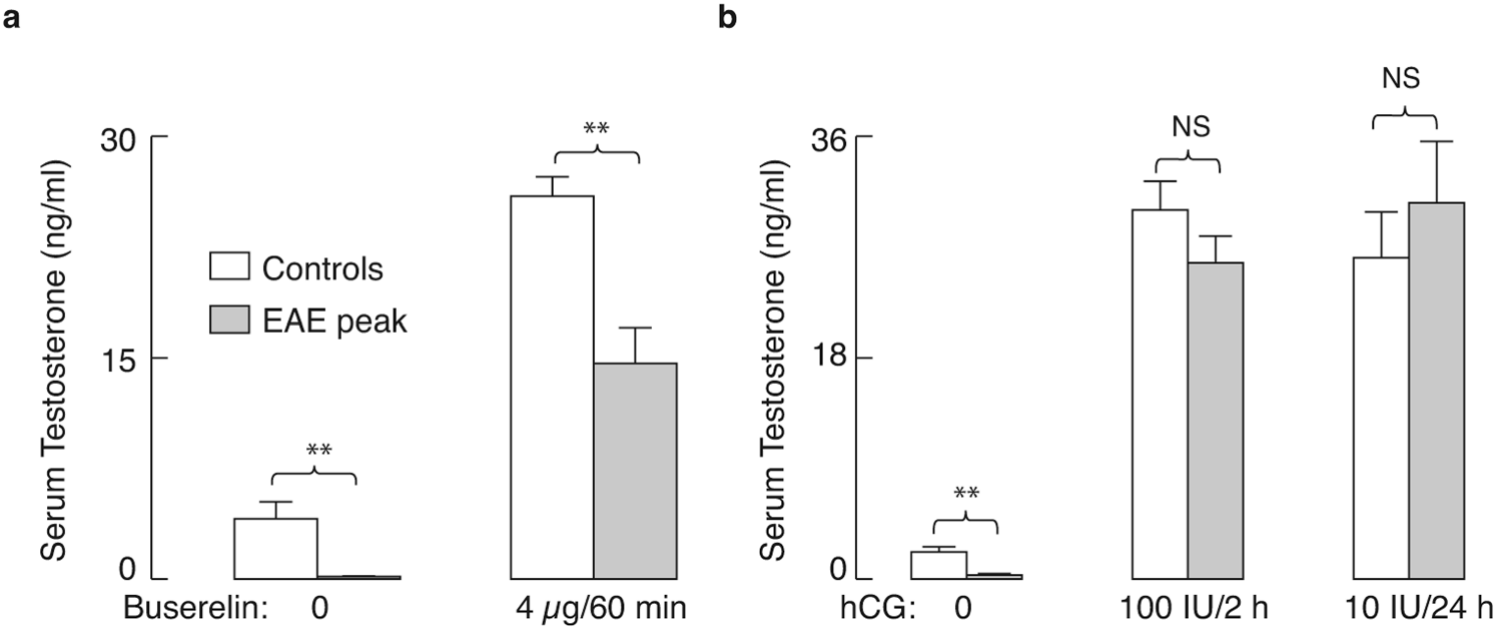
Effects of EAE on basal and stimulated serum testosterone levels. (**a**) Controls (white bars) and peak EAE animals (gray bars) were injected intraperitoneally with saline (left panel) or 4 µg buserelin acetate, a GnRHR agonist (right panel) and blood was collected 1 h after injection. (**b**) Controls (white bars) and peak EAE rats (gray bars) were injected subcutaneously with saline (left panel), 100 IU hCG for 2 h (middle panel), or 10 IU hCG for 24 h (right panel). The results are presented as mean ± SEM (n = 6 animals/group). Statistical evaluation between pairs was determined by Mann-Whitney test. * *P* < 0.05, ** *P* < 0.01, NS, nonsignificant.The figure was made using KaleidaGraph (version 4.1.0; http://www.synergy.com) and Illustrator (version 25.1.0; http://www.adobe.com).

We hypothesized that the main factor responsible for the decrease in serum testosterone during EAE is the deficiency of LH, which causes the down-regulation of steroidogenic pathway components. To test the status of the pituitary gland in EAE animals, rats were treated with intraperitoneal injection of buserelin acetate, a GnRHR agonist, aimed at activating these receptors in pituitary gonadotrophs and stimulating LH secretion. Testicular responsiveness in terms of serum testosterone levels is shown in Fig. 5a, right; there was a significant increase in testosterone levels in the control group (*P* < 0.05) and peak EAE group (*P* < 0.01) 1 h after buserelin acetate injection. However, a significant difference in response amplitude between those two groups was observed (*P* < 0.01). The poorer response of diseased animals to buserelin acetate is in line with our recent findings that 1 h after this treatment, LH recovers in male rats at the peak of EAE, but does not reach the response amplitude of control animals^9^.

We also injected subcutaneously two different doses of hCG to control and peak EAE groups of rats to evaluate their responsiveness to this LHR agonist. Two hours after treatment with 100 IU hCG, serum testosterone was significantly elevated both in control and EAE group (Fig. 5b, middle). Injection of 10 IU hCG also significantly increased serum testosterone in both groups of animals after 24 h (Fig. 5b, right). In addition, no difference in testosterone production could be observed when controls were compared to peak EAE animals at either time-point of hCG treatment, meaning that the testes remained equally responsive for hCG in healthy and diseased animals. In both experiments, untreated peak EAE rats showed a significant (*P* < 0.01) reduction in serum testosterone levels compared with untreated controls (Fig. 5a, left and 5b, left). These results indicate that the secretory pathway in the pituitary gonadotrophs is affected, leading to a decrease in endogenous LH release and subsequent down-regulation of LH-dependent testicular functions.

### The hCG-induced expression of steroidogenic pathway genes was the same in control and diseased animals

**Figure 6:**
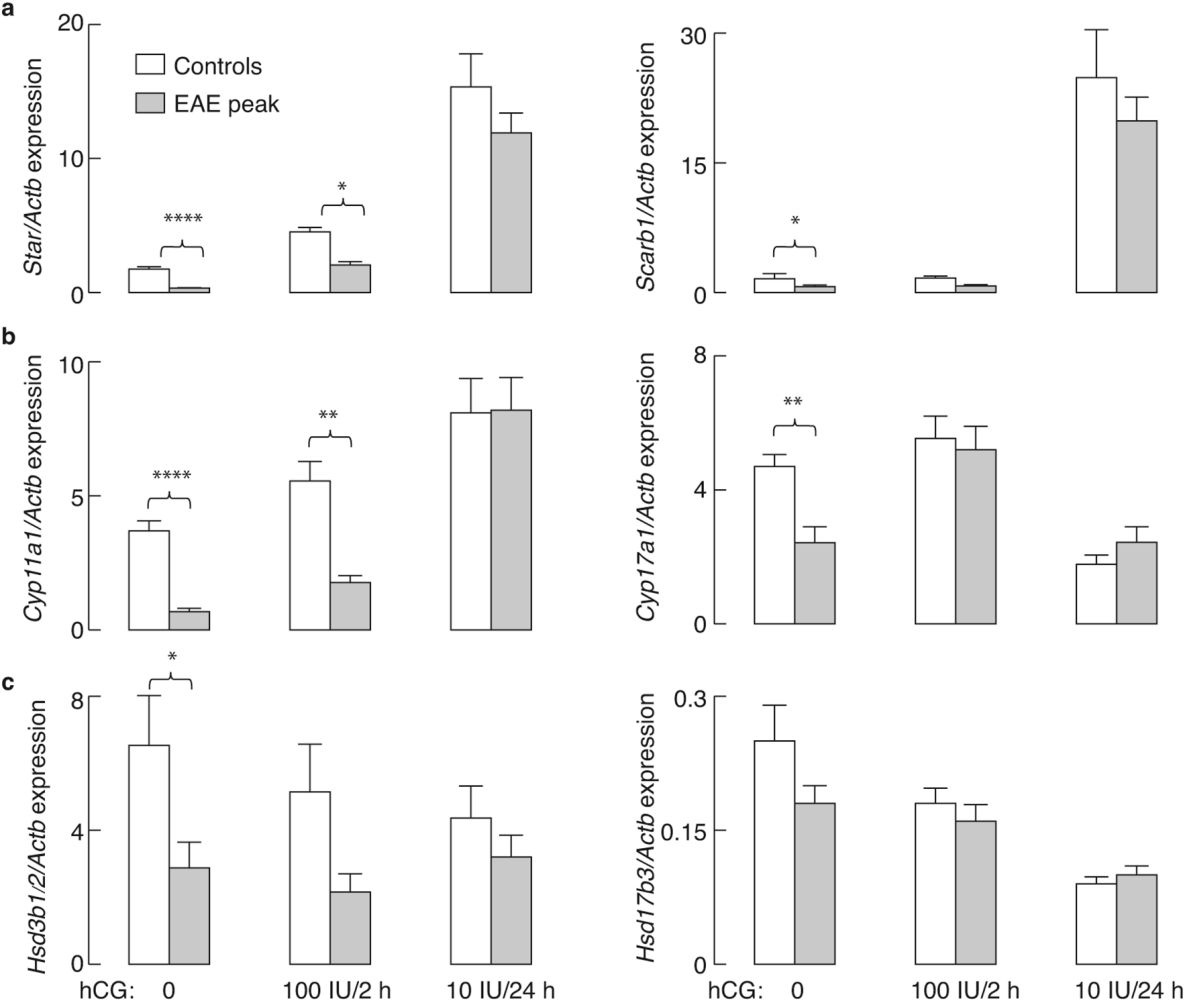
Expression of steroidogenic genes in testicular interstitial cells in control and peak EAE groups of saline- and hCG-treated rats. Control and peak EAE rats were treated with a subcutaneous injection of saline, 100 IU hCG for 2 h, or 10 IU hCG for 24 h. qRT-PCR analysis of rat interstitial cells: (**a**) *Star* (left) and *Scarb1* (right)*;* (**b**) *Cyp11a1* (left) and *Cyp17a1* (right); (**c**) *Hsd3b1/2* (left) and *Hsd17b3* (right). The results are presented as mean ± SEM (n = 6 animals/group). The presented statistical significance was determined by Mann–Whitney test for control and peak EAE groups in each treatment. The results are presented as mean ± SEM. *P < 0.05, **P < 0.01, ***P < 0.001, ****P < 0.0001. The figure was made using KaleidaGraph (version 4.1.0; http://www.synergy.com) and Illustrator (version 25.1.0; http://www.adobe.com).

As shown in Fig. 2, the expression of *Scarb1*, *Star*, *Cyp11a1*, *Cyp17a1*, and *Hsd3b1/2* was significantly reduced at the peak of EAE. The same conclusion was made in animals at the peak of the disease used to study effects of hCG treatments (Fig. 6, hCG 0 treatment).

Two hours after 100 IU hCG treatment, the expression of *Star* (Fig. 6a, left) and *Cyp17a1* (Fig. 6b, right) was increased several-fold in peak EAE animals compared to their untreated peers. As expected, the effects of 10 IU hCG were more pronounced 24 h after the injection; *Star* (Fig. 6a, left), *Cyp11a1* (Fig. 6b, left) and *Scarb1* (Fig. 6a, right) mRNA levels were up-regulated in both control and peak EAE groups. In addition, the response of these genes to hCG treatment did not differ significantly between healthy and EAE animals. *Hsd17b3* expression was unchanged after 2 h of 100 IU hCG but significantly decreased 24 h after 10 IU hCG in both control and peak EAE animals (Fig. 6c, right). No difference was observed between control and peak EAE groups at either time-point after hCG injection in *Cyp17a1* mRNA levels, but with reduced expression of this enzyme gene in control 24 h after treatment (Fig. 6b, right). The expression profile of *Hsd3b1/2* was unaltered by hCG (Fig. 6c, left). These results imply that, during EAE, impaired testicular steroidogenesis arises as a consequence of decreased LH release.

## Discussion

We here aimed to clarify the phenomenon of impaired testicular steroidogenesis during EAE. Although the effects of EAE on steroidogenesis are pronounced, our results show that there are no changes in testicular weight or interstitial cell number. Any gross damage to the testicular tissue is also excluded by the fact that all the tested parameters recover spontaneously at the end of the disease. The presence of spermatocytes in the seminiferous tubules of peak EAE animals, shown here and observed earlier^9^, indicates that this acute disease does not significantly affect spermatogenesis. This is consistent with extensive literature data showing that spermatogenesis occurs even when serum testosterone levels are low^26,27^. As a monophasic disease, EAE does not last long enough to produce grossly visible changes in testicular morphology. It also does not reproduce a lasting testosterone deficiency seen in MS. In this regard, EAE model is not adequate to study the long-term effects of steroidogenesis down-regulation.

On the other hand, EAE appears to be a good model for studying the mechanisms that lead to suppressed steroidogenesis during MS. Our results show that *Insl3* mRNA expression is reduced at the peak of EAE in the testes. This implies that the dynamics of LC differentiation and proliferation and their functional capacity can be disrupted^28^ without affecting the total number of cells, or that these cells may be going through a transformation process^29^ during the disease. Consistent with these findings, we report here for the first time a decrease in gene and protein expression of steroidogenic pathway components in testicular interstitial cells during the onset/peak of EAE.

LCs are the only testicular cells that carry LHR^30^ and the expression and functionality of these receptors is primarily affected by LH deficiency^31,32^, but this does not necessarily indicate a decrease in the number of LCs in the testes^31,33,34^. Here we show that the *Lhcgr* expression is reduced during EAE. We do not currently know whether EAE affects the LHR number, but since hCG stimulation elicits clear responses, it is likely that this number is not severely affected.

A decrease in *Star*, *Cyp11a1*, *Cyp17a1* and *Hsd3b1/2* mRNA expression, and StAR and CYP11A1 protein expression in interstitial testicular cells during the onset/peak of EAE can also be attributed to LH deficiency, which may directly affect the expression of steroidogenic pathway components and/or indirectly by the loss of endoplasmic reticulum and mitochondrial volume and surface area^13,31,33–38^. Accordingly, the recovery of serum LH levels towards the end of the disease is accompanied by a recovery of the expression of steroidogenic components (both gene and protein), and consequently of testosterone secretion. Interestingly, while *Hsd3b1/2* expression is significantly reduced, protein levels remain disease-resistant. It has been suggested that HSD3B is constantly present in LCs independent of cAMP treatment^38^, probably due to the high constitutive mRNA expression of this enzyme in rodents^37,39^.

We investigated the possibility that EAE causes an inflammatory environment in the testis, leading to suppression of testosterone synthesis. Most types of immune cells can be found in the interstitial spaces of mammalian testes, but macrophages represent more than 20% of interstitial cells^40^. Testicular macrophages are the main immune cell population involved in the regulation of steroidogenesis and Sertoli cell activity, and their number changes in various pathological states^41–43^. However, our analysis implies that tissue distribution and the number of “newly arrived” CD68-positive macrophages do not change in EAE. Also, hematoxylin counterstained sections do not show mononuclear cell infiltration. These results, together with the low expression of proinflammatory cytokine genes in the testis, indicate the absence of a local inflammatory response during EAE.

It is well established that inflammatory cytokines can inhibit cAMP-induced steroidogenesis^38,44,45^. For example, a single intracerebroventricular injection of IL-1β blunted the effects of hCG stimulation on testosterone production^46^. Similarly, LPS-induced levels of TNFα and IL-6 suppressed testicular response to hCG in rats^47,48^. In our experiments, however, EAE and control animals respond similarly to hCG treatment, further confirming that a decrease in circulating gonadotropins, rather than an increase in circulating cytokines, is responsible for the decrease in testosterone during EAE.

Our further research revealed response patterns in control and EAE animals after hCG application. In terms of testosterone production, diseased animals responded to hCG very similarly to their healthy counterparts. This is not surprising because 2 h^49^ or two consecutive days^34^ of hCG treatment partially restored testosterone production even after chronic (4–5 weeks) LH deficiency caused by hypophysectomy. Thus, regressed LCs can rapidly regain steroidogenic activity. However, LH deficiency is not as pronounced in EAE as in hypophysectomized animals. It should also be noted that buserelin acetate induced testosterone production within one hour, although not with equal efficiency. This is consistent with the lower response of EAE animals to buserelin acetate, in terms of LH secretion^9^.

Also, the steroidogenic pathway genes’ expression was similar after hCG treatment in control and diseased animals. Rapid induction of *Star* expression is consistent with the finding that trophic hormones stimulate its expression within 30 min^13,50^. In contrast, we note here that *Cyp11a1* expression increases more slowly than *Star* in response to LHR stimulation, in line with other reports^51^.

Interestingly, longer hCG stimulation down-regulated *Cyp17a1* expression, most likely due to suppression by endogenously produced testosterone, as is the case in cultured LCs and MA-10 cells^37,52,53^. The lack of noticeable effects of hCG on *Hsd3b* expression is in concordance with findings that cAMP stimulates *Hsd3b* expression only when steroid hormone synthesis is inhibited in primary mouse LCs^37^. In addition, the fact that hCG stimulation induced *Scarb1* expression is consistent with findings suggesting that LH is responsible for the induction of this cholesterol pathway in LCs^54,55^.

Patients with MS may have a chronic testosterone deficiency, which can be reflected in sexual dysfunction, fertility problems and the course of the disease itself. Testosterone therefore remains of interest as a therapeutic agent in MS; currently one study is recruiting patients for the testosterone undecanoate trial^56^. In a small cohort of MS patients, testosterone gel (applied daily for one year) has been shown to improve cerebral gray matter density^57^. Although we show here that hCG can restore testosterone production, other studies are needed to show whether gonadotropins can be used as therapeutics in EAE/MS to induce sustained testosterone production and alter the course of the disease. In this respect, hCG should not be the gonadotropin of choice due to its long half-life and subsequent LHR desensitization, and we are currently developing strategies for more efficient treatments.

In conclusion, here we described for the first time the effects of temporal progression of EAE on the expression profile of testicular steroidogenic pathway. In addition, we show the testicular response to direct hCG and indirect buserelin acetate stimulation at the peak of EAE, in terms of testosterone production and the absence of testicular inflammation. These observations strongly support the hypothesis that EAE does not directly affect the testes and that the pituitary LH deficiency is the major cause for low testosterone during the disease. Furthermore, the changes that occur in the testes during EAE are reversible. This and similar studies will greatly contribute to our current knowledge and understanding the link between testosterone and this disease, while paving the way to future possibilities for gonadotropin treatments and restoration of endogenous testosterone production in men with MS related testosterone deficiency.

## Supplementary Information

Supplementary information.

## References

[1] Harbo HF, Gold R, Tintoré M (2013). Sex and gender issues in multiple sclerosis. Ther. Adv. Neurol. Disord..

[2] Whitacre CC, Reingold SC, O'Looney PA (1999). A gender gap in autoimmunity. Science.

[3] Chitnis T (2018). The role of testosterone in MS risk and course. Multiple Scler. (Houndmills, Basingstoke, England).

[4] Foster SC, Daniels C, Bourdette DN, Bebo BF (2003). Dysregulation of the hypothalamic-pituitary-gonadal axis in experimental autoimmune encephalomyelitis and multiple sclerosis. J. Neuroimmunol..

[5] Safarinejad MR (2008). Evaluation of endocrine profile, hypothalamic-pituitary-testis axis and semen quality in multiple sclerosis. J. Neuroendocrinol..

[6] Wei T, Lightman SL (1997). The neuroendocrine axis in patients with multiple sclerosis. Brain.

[7] Bove R (2014). Low testosterone is associated with disability in men with multiple sclerosis. Multiple Scler. (Houndmills, Basingstoke, England).

[8] Macció DR, Calfa G, Volosín M, Roth GA (2004). Serum testosterone and corticosterone levels in acute experimental autoimmune encephalomyelitis (EAE) in male Wistar rats. Neuro Endocrinol. Lett..

[9] Milosevic A (2020). The sex-specific patterns of changes in hypothalamic-pituitary-gonadal axis during experimental autoimmune encephalomyelitis. Brain Behav. Immun..

[10] Dufau ML, Catt KJ (1978). Gonadotropin receptors and regulation of steroidogenesis in the testis and ovary. Vitam. Horm..

[11] Connelly MA, Williams DL (2003). SR-BI and cholesterol uptake into steroidogenic cells. Trends Endocrinol. Metab..

[12] Clark BJ, Wells J, King SR, Stocco DM (1994). The purification, cloning, and expression of a novel luteinizing hormone-induced mitochondrial protein in MA-10 mouse Leydig tumor cells. Characterization of the steroidogenic acute regulatory protein (StAR). J. Biol. Chem..

[13] Selvaraj V, Stocco DM, Clark BJ (2018). Current knowledge on the acute regulation of steroidogenesis. Biol. Reprod..

[14] Payne AH, Hales DB (2004). Overview of steroidogenic enzymes in the pathway from cholesterol to active steroid hormones. Endocr. Rev..

[15] Bremer AA, Miller WL, Alfredo U-A, Michael Conn P (2014). Cellular Endocrinology in Health and Disease.

[16] Collongues N, Patte-Mensah C, De Seze J, Mensah-Nyagan AG, Derfuss T (2018). Testosterone and estrogen in multiple sclerosis: From pathophysiology to therapeutics. Expert Rev. Neurother..

[17] Macció DR, Calfa G, Roth GA (2005). Oral testosterone in male rats and the development of experimental autoimmune encephalomyelitis. NeuroImmunoModulation.

[18] Gubbels Bupp MR, Jorgensen TN (2018). Androgen-induced immunosuppression. Front. Immunol..

[19] Palaszynski KM, Loo KK, Ashouri JF, Liu HB, Voskuhl RR (2004). Androgens are protective in experimental autoimmune encephalomyelitis: implications for multiple sclerosis. J. Neuroimmunol..

[20] Hodgson YM, de Kretser DM (1982). Serum Testosterone response to single injection of hCG ovine-LH and LHRH in male rats. Int. J. Androl..

[21] Tremblay Y, Belanger A (1985). Changes in plasma steroid levels after single administration of hCG or LHRH agonist analogue in dog and rat. J. Steroid Biochem..

[22] Anakwe OO, Murphy PR, Moger WH (1985). Characterization of beta-adrenergic binding sites on rodent Leydig cells. Biol. Reprod..

[23] Andric SA, Janjic MM, Stojkov NJ, Kostic TS (2007). Protein kinase G-mediated stimulation of basal Leydig cell steroidogenesis. Am. J. Physiol.-Endocrinol. Metab..

[24] Jakovljevic M (2017). Down-regulation of NTPDase2 and ADP-sensitive P2 purinoceptors correlate with severity of symptoms during experimental autoimmune encephalomyelitis. Front. Cell. Neurosci..

[25] Herrmann M, Scholmerich J, Straub RH (2002). Influence of cytokines and growth factors on distinct steroidogenic enzymes in vitro: A short tabular data collection. Ann. N. Y. Acad. Sci..

[26] Sharpe RM, Donachie K, Cooper I (1988). Re-evaluation of the intratesticular level of testosterone required for quantitative maintenance of spermatogenesis in the rat. J. Endocrinol..

[27] Zirkin BR, Santulli R, Awoniyi CA, Ewing LL (1989). Maintenance of advanced spermatogenic cells in the adult rat testis: Quantitative relationship to testosterone concentration within the testis. Endocrinology.

[28] Ivell R, Wade JD, Anand-Ivell R (2013). INSL3 as a biomarker of Leydig cell functionality. Biol. Reprod..

[29] Bay K (2005). Insulin-like factor 3 serum levels in 135 normal men and 85 men with testicular disorders: Relationship to the luteinizing hormone-testosterone axis. J. Clin. Endocrinol. Metab..

[30] Catt KJ, Dufau ML, Lutz B, O’Malley BW (1978). Receptors and Hormone Action.

[31] Russell LD (1992). Structural changes in rat Leydig cells posthypophysectomy: A morphometric and endocrine study. Endocrinology.

[32] Hanour F, Sanchez P, Cathiard AM, Saez JM (1978). Gonadotropin receptor regulation in hypophysectomized rat Leydig cells. Biochem. Biophys. Res. Commun..

[33] Keeney DS, Mendis-Handagama SM, Zirkin BR, Ewing LL (1988). Effect of long term deprivation of luteinizing hormone on Leydig cell volume, Leydig cell number, and steroidogenic capacity of the rat testis. Endocrinology.

[34] Stocco DM, Teerds KJ, van Noort M, Rommerts FF (1990). Effects of hypophysectomy and human chorionic gonadotrophin on Leydig cell function in mature rats. J. Endocrinol..

[35] Wing TY, Ewing LL, Zirkin BR (1984). Effects of luteinizing hormone withdrawal on Leydig cell smooth endoplasmic reticulum and steroidogenic reactions which convert pregnenolone to testosterone. Endocrinology.

[36] Klinefelter GR, Hall PF, Ewing LL (1987). Effect of luteinizing hormone deprivation in situ on steroidogenesis of rat Leydig cells purified by a multistep procedure. Biol. Reprod..

[37] Payne AH, Sha LL (1991). Multiple mechanisms for regulation of 3 beta-hydroxysteroid dehydrogenase/delta 5––delta 4-isomerase, 17 alpha-hydroxylase/C17-20 lyase cytochrome P450, and cholesterol side-chain cleavage cytochrome P450 messenger ribonucleic acid levels in primary cultures of mouse Leydig cells. Endocrinology.

[38] Payne AH, Youngblood GL (1995). Regulation of expression of steroidogenic enzymes in Leydig cells. Biol. Reprod..

[39] Keeney DS, Mason JI (1992). Expression of testicular 3 beta-hydroxysteroid dehydrogenase/delta 5–4-isomerase: Regulation by luteinizing hormone and forskolin in Leydig cells of adult rats. Endocrinology.

[40] Hedger MP (2002). Macrophages and the immune responsiveness of the testis. J. Reprod. Immunol..

[41] Frungieri MB (2002). Number, distribution pattern, and identification of macrophages in the testes of infertile men. Fertil. Steril..

[42] Rival C (2008). Functional and phenotypic characteristics of testicular macrophages in experimental autoimmune orchitis. J. Pathol..

[43] Gerdprasert O (2002). The response of testicular leukocytes to lipopolysaccharide-induced inflammation: Further evidence for heterogeneity of the testicular macrophage population. Cell Tissue Res..

[44] Orava M, Voutilainen R, Vihko R (1989). Interferon-gamma inhibits steroidogenesis and accumulation of mRNA of the steroidogenic enzymes P450scc and P450c17 in cultured porcine Leydig cells. Mol. Endocrinol..

[45] Guzman C, Hernández-Bello R, Morales-Montor J (2010). Regulation of steroidogenesis in reproductive, adrenal and neural tissues by cytokines. Open Neuroendocrinol. J..

[46] Turnbull AV, Rivier C (1997). Inhibition of gonadotropin-induced testosterone secretion by the intracerebroventricular injection of interleukin-1 beta in the male rat. Endocrinology.

[47] Rivier C (2002). Inhibitory effect of neurogenic and immune stressors on testosterone secretion in rats. NeuroImmunoModulation.

[48] O'Bryan MK, Schlatt S, Phillips DJ, de Kretser DM, Hedger MP (2000). Bacterial lipopolysaccharide-induced inflammation compromises testicular function at multiple levels in vivo. Endocrinology.

[49] Hodgson YM, de Kretser DM (1985). Testosterone response of cryptorchid and hypophysectomized rats to human chorionic gonadotrophin (hCG) stimulation. Aust. J. Biol. Sci..

[50] Manna PR, Wang XJ, Stocco DM (2003). Involvement of multiple transcription factors in the regulation of steroidogenic acute regulatory protein gene expression. Steroids.

[51] Lavoie HA, King SR (2009). Transcriptional regulation of steroidogenic genes: STARD1, CYP11A1 and HSD3B. Exp. Biol. Med..

[52] Hales DB, Sha LL, Payne AH (1987). Testosterone inhibits cAMP-induced de Novo synthesis of Leydig cell cytochrome P-450(17 alpha) by an androgen receptor-mediated mechanism. J. Biol. Chem..

[53] Burgos-Trinidad M (1997). Repression of cAMP-induced expression of the mouse P450 17 alpha-hydroxylase/C17–20 lyase gene (Cyp17) by androgens. Mol. Endocrinol..

[54] Landschulz KT, Pathak RK, Rigotti A, Krieger M, Hobbs HH (1996). Regulation of scavenger receptor, class B, type I, a high density lipoprotein receptor, in liver and steroidogenic tissues of the rat. J. Clin. Investig..

[55] Reaven E, Zhan L, Nomoto A, Leers-Sucheta S, Azhar S (2000). Expression and microvillar localization of scavenger receptor class B, type I (SR-BI) and selective cholesteryl ester uptake in Leydig cells from rat testis. J. Lipid Res..

[56] Metzger-Peter K (2020). The TOTEM RRMS (Testosterone treatment on neuroprotection and Myelin repair in relapsing remitting multiple sclerosis) trial: Study protocol for a randomized, double-blind, placebo-controlled trial. Trials.

[57] Kurth F (2014). Neuroprotective effects of testosterone treatment in men with multiple sclerosis. NeuroImage. Clin..

