# Supplementary material for: Testicular steroidogenesis is suppressed during experimental autoimmune encephalomyelitis in rats
Source: Sci Rep. 2021 Apr 26;11:8996. doi: 10.1038/s41598-021-88305-5 (PMC8076254; doi:10.1038/s41598-021-88305-5)
Supplement: Supplementary file 1 — Supplementary information. [file 41598_2021_88305_MOESM1_ESM.pdf]

Testicular steroidogenesis is suppressed during experimental autoimmune encephalomyelitis in rats

Ana Milosevic[1], Ivana Bjelobaba[1], Iva D. Bozic[1], Irena Lavrnja[1], Danijela Savic[1], Katarina Tesovic[1], Marija Jakovljevic[1], Stanko S. Stojilkovic[2], Marija M. Janjic[1,*]

[1]Institute for Biological Research "Siniša Stanković"- National Institute of Republic of Serbia, University of Belgrade, Belgrade, Serbia

[2]Section on Cellular Signaling, *Eunice Kennedy Shriver* National Institute for Child Health and Human Development, National Institutes of Health, Bethesda, MD, USA

Running Head: Testicular steroidogenesis during EAE

Word Count:

Main Text = 3159

Methods = 1534

*** Correspondence:**

Marija Janjic, Department for Neurobiology, Institute for Biological Research "Siniša Stanković"- National Institute of Republic of Serbia, Bulevar despota Stefana 142, 11000 Belgrade, Serbia.

Phone: +381 11 2078 340; Fax: +381 11 2078 433; E-mail: marija.janjic@ibiss.bg.ac.rs

Disclosure Statement: The authors have nothing to disclose.

Key words: EAE, multiple sclerosis, testes, steroidogenesis, testosterone, LH.

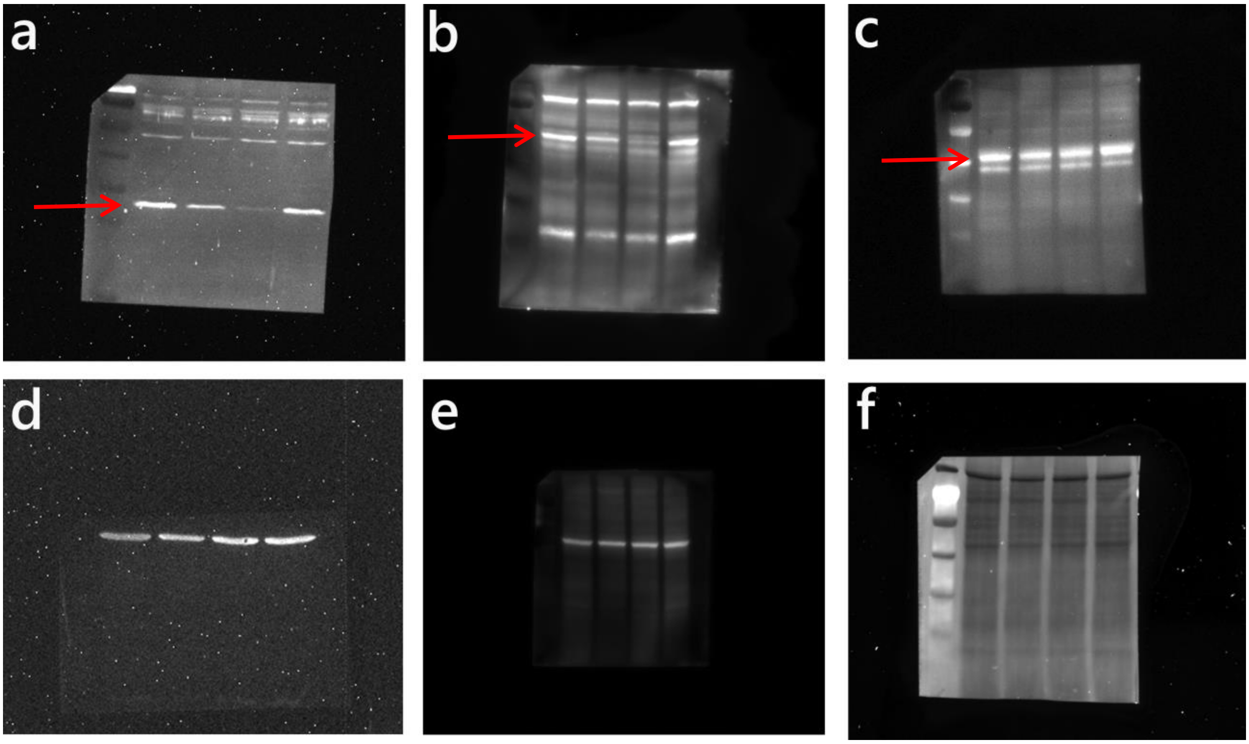

Supplementary figure. Full unprocessed blots shown in Figure 3. a. StAR (~30 kDa), **b**. CYP11A1 (~50 kDa), **c**. HSD3B (~45 kDa), **d**. β-actin (for StAR, 42 kDa), **e**. β-actin (for CYP11A1, 42 kDa), **f**. total protein stain (for HSD3B).
